# Association between IL-1A and IL-1B gene polymorphisms with peri-implantitis in a Portuguese population—a pilot study

**DOI:** 10.7717/peerj.13729

**Published:** 2022-07-14

**Authors:** José Maria Cardoso, Ana Clara Ribeiro, Constança Palos, Luis Proença, Susana Noronha, Ricardo Castro Alves

**Affiliations:** 1 Centro de Investigação Interdisciplinar Egas Moniz (CiiEM), Instituto Universitário Egas Moniz, Almada, Portugal; 2 Periodontology Department, Instituto Universitário Egas Moniz, Almada, Portugal; 3 Molecular Biology Laboratory, Instituto Universitário Egas Moniz, Almada, Portugal; 4 Periodontology Department, Faculdade de Medicina Dentária da Universidade de Lisboa, Lisboa, Portugal

**Keywords:** Genetics, Peri-implantitis, Interleukin-1, Single-nucleotide polymorphism, Interleukin-1-genotype, Genetic polymorphisms, Peri-implant disease

## Abstract

**Background:**

Scientific evidence indicates that biological complications in dental implants tend to be concentrated in a subset of individuals, which seems to imply that the host response may play a determining role in implant success. Over the last few decades, several polymorphisms have been studied. Polymorphisms in the interleukin (IL) 1 gene cluster have been associated with periodontitis. There are some similar features in the sequence of immunopathological events in peri-implant and periodontal infections. We aimed to investigate if individuals carrying the genetic single nucleotide polymorphism (SNP) in the IL-1A (rs1800587) and IL-1B (rs1143634) genes are more susceptible to develop peri-implantitis.

**Methods:**

A cross-sectional analytic pilot study was conducted in 20 Caucasian Portuguese subjects divided into two groups: 10 subjects with peri-implantitis and 10 subjects with peri-implant health (control group). Samples containing cells from the buccal mucosa were stored at −20 °C and later submitted to the DNA extraction process. Genetic analysis was performed using the polymerase chain reaction (PCR) and restriction fragment length polymorphism (RFLP) analysis. Data were analyzed by using descriptive and inferential statistical methodologies.

**Results:**

For the IL-1A (−889) gene polymorphism, it was observed that the mutated allele was present in a higher percentage in the peri-implantitis group compared to the control group (30% vs 15% respectively, Fisher’s exact test, *p* = 0.45). For the IL-1B (+3954) gene polymorphism, it was also observed that the altered allele was present in a higher percentage in the disease group compared to the control group (35% vs 10% respectively, Fisher’s exact test, *p* = 0.13). The positive genotype (at least one allele with nucleotide sequence changed in both genes) was detected in six patients, five belonging to the disease group and one to the health group.

**Conclusions:**

Regarding IL-1 gene polymorphisms, there was no statistically significant difference between the health and disease group, however a trend should be highlighted, showing a potential link between the IL-1 genotype and peri-implantitis. More studies are needed to clarify the role of genetic polymorphisms in the development of peri-implantitis.

## Introduction

Peri-implantitis is a pathological condition occurring in tissues around dental implants, characterized by inflammation in the peri-implant mucosa and progressive loss of supporting bone (Lindhe, Meyle & Group D of European Workshop on Periodontology, 2008; Lang, Berglundh & Working Group 4 of Seventh European Workshop on Periodontology, 2011). Atieh et al. (2013) published a meta-analysis and concluded that the prevalence of peri-implantitis was 9.6% when assessed at the implant level and 18.8% when calculated per patient. In smokers, a high-risk group according to current evidence, the prevalence rates obtained for peri-implantitis increased to 36.3%. More recently, Lee et al. (2017) conducted a systematic review and meta-analysis that included forty-seven articles, and concluded that the mean prevalence of peri-implantitis, at implant and subject level was 9.25% and 19.83%, respectively.

Scientific evidence indicates that biological complications in dental implants tend to be concentrated in a subset of individuals, which seems to imply that the host response might be a key determinant in the development of peri-implantitis (Fransson et al., 2010; Chen & Zhao, 2019). The host immune system reacts to bacterial invasion by producing inflammatory mediators. The pro-inflammatory cytokine interleukin-1 (IL-1) is considered a major mediator of chronic inflammatory diseases, playing a crucial role in the process of destruction of the extracellular matrix of connective tissue and bone resorption (Dinarello, 1994). It is also a primary activator of chemotactic cytokines and increases the expression of adhesion molecules that facilitate the migration of leukocytes. Additionally, IL-1 stimulates the production of other pro-inflammatory mediators such as tumor necrosis factor alpha (TNF-α) and prostaglandin E2 (PGE2) (Alassy, Parachuru & Wolff, 2019). Several studies have shown high levels of IL-1 in peri-implant crevicular fluid in patients with peri-implant infections (Salcetti et al., 1997; Schminke et al., 2015).

The IL-1A (rs1800587) and IL-1B (rs1143634) genes have been described as being able to regulate the production of IL-1α and IL-1β, respectively (Nicklin, Weith & Duff, 1994). They are located close to each other, on the long arm of chromosome 2, specifically at position 2q13, and are considered potential candidates for genetic markers in peri-implantitis, since the cytokines they encode play a crucial role in the development of the inflammatory response (Greenstein & Hart, 2002).

Kornman et al. (1997) showed for the first time that the combined presence of allele 2 (allele with nucleotide sequence changed) of the IL-1A gene at nucleotide position –889 (IL-1A-889T) and the allele 2 of IL-1B gene at nucleotide position + 3954 (IL-1B+3954T) was associated with an increased risk for developing severe periodontitis in nonsmoking Caucasian patients. The replacement of the cytosine base by a thymine occurred in the promoter region of the IL-1A gene and in exon 5 of the IL-1B gene. The presence of at least one allele 2 at the IL-1A-889 locus and at least one allele 2 at the IL-1B+3954 locus is designated IL-1 positive genotype. These polymorphisms of IL-1A-889T and IL-1B+3954T are single nucleotide polymorphisms (SNP’s).

The sequence of immunopathological events in peri-implant infections has some similarities to periodontal infections (Belibasakis, 2014). However, when compared to experimentally induced periodontitis, lesions associated with experimental peri-implantitis demonstrate larger inflammatory cell infiltrates and more rapid and pronounced bone loss. Similar to periodontitis, the lesions at peri-implantitis sites were also dominated by plasma cells and lymphocytes, but characterized by larger proportions of polymorphonuclear leukocytes and macrophages (Schwarz et al., 2018).

There are some scientific research studies that have analyzed the role of the polymorphisms IL-1A-889T and IL-1B+3954T in host response in the development of biological complications, in dental implants (Feloutzis et al., 2003; Shimpuku et al., 2003; Gruica et al., 2004; Laine et al., 2006; Lachmann et al., 2007; Hamdy & Ebrahem, 2011; Vaz et al., 2012; He et al., 2020). Some of these studies observed that there is no relationship between the prevalence of these polymorphisms and peri-implantitis (Feloutzis et al., 2003; Shimpuku et al., 2003; Gruica et al., 2004; Laine et al., 2006; Lachmann et al., 2007). However, in a subgroup analysis it is observed that the IL-1 positive genotype in heavy smokers corresponded to a higher risk for the development of more severe inflammatory complications and increased peri-implant bone loss (Feloutzis et al., 2003; Gruica et al., 2004).

Lachmann et al. (2007) evaluated the association between peri-implant crevicular fluid volume, crevicular inflammatory mediators’ concentrations and the IL-1 positive genotype and the presence of peri-implantitis. In patients with peri-implantitis, no significant influence of altered genotype was observed.

In a study by He et al. (2020) with a sample of 318 Chinese patients, the authors observed that subjects carrying the T allele of IL-1A -889 and IL-1B +3954 had a significant 2.27–2.47-fold and 1.9–1.99-fold increased risk of peri-implantitis, respectively, when using the CC genotype as reference.

Fourmousis & Vlachos (2019) performed a review in which concluded that the identification of genetic biomarkers associated with peri-implantitis risk could be valuable in daily clinical practice, however, no robust conclusions could be drawn from the current literature.

The number of studies in the literature evaluating this association is limited and some of these does not refer to confounding variables such as periodontal condition, ethnicity and smoking status. The definition of peri-implantitis also varies between studies. The inequality of these studies’ design necessitates the conduction of further studies using proper methodologies and from different ethnic groups (Fourmousis & Vlachos, 2019).

A clarification of the genetic basis associated with peri-implant pathology could be used to predict peri-implantitis occurrence and to improve treatment and monitoring of patients with dental implants (Laine et al., 2006).

According to the available evidence it is not possible to reach robust conclusions about the influence of IL-1 polymorphisms on the development of peri-implantitis. Furthermore, there are few studies that evaluate this possible relationship in certain ethnic groups, namely in Caucasians from southern Europe.

In the present study, we aimed to investigate the possible association of IL-1A- 889T and IL-1B+ 3954T polymorphisms with the peri-implantitis in a Portuguese cohort. We hypothesize that individuals carrying the T allele in the IL-1A-889 and IL-1B +3954 genes are more susceptible to develop peri-implantitis in comparison with individuals that don’t have these polymorphisms.

## Materials & Methods

### Study design and population

The study was conducted according to the principles of the Helsinki Declaration (reviewed in 2013). Appropriate measures were taken for the protection of patient data. The study was approved by the Ethics Committee of Instituto Universitário Egas Moniz on July 24th, 2019 (process number 790).

The study included two groups, the disease group consisting of ten patients with peri-implantitis (bleeding and/ or suppuration on probing, probing depth equal or greater than six mm and bone loss equal to or greater than three mm) and the control group formed by ten patients with peri-implant health (peri implant mucosa without inflammatory signs and absence of peri-implant bone loss).

The definitions of peri-implantitis, peri-implant health, periodontal health and periodontitis case were in accordance with the new classification of periodontal and peri-implant diseases (American Academy of Periodontology - AAP and European Federation of Periodontology - EFP 2018) (Berglundh et al., 2018; Chapple et al., 2018; Papapanou et al., 2018).

The convenience sample was obtained from patients referred to periodontal treatment at the Periodontology Department of Egas Moniz Dental Clinic (EMDC), between July of 2020 and February of 2021. All possible candidates received a screening questionnaire and if the patient’s medical history was in accordance with study inclusion criteria, and if they agreed to participate, informed written consent was obtained.

The inclusion criteria were: patients with Caucasian parents and grandparents; patients with dental implants in function for at least one year; patients with peri-implantitis diagnosis; patients who agree to participate in the study and sign informed consent. The exclusion criteria were: patients with any systemic condition that affects the immune system; patients who have taken antibiotics or anti-inflammatory drugs on a chronic basis in the last six months; pregnant women; patients who have undergone peri-implant treatment in the area to be evaluated.

### Socio-demographic and clinical variables

Data were gathered on age, sex, smoking habit (yes or not) and history of periodontitis.

For patients with suspected periodontitis a full periodontal examination (six locations per tooth with a CP12 graduated periodontal probe) was performed recording probing pocket depth, bleeding on probing, gingival margin and tooth mobility.

A peri-implant probing examination (six locations with a CP12 graduated periodontal probe) was performed as usual at periodontology appointments. The presence of bleeding on probing or pus were also evaluated. An implant control radiograph was taken in cases where there was a probing depth more than six mm (parallelometric technique).

All clinical data was collected by the same examiner (J.M.C.). Prior to the study, a training and calibration exercise was performed for probing pocket depth (PPD) measurements using volunteer patients not included in the study. The calibration exercise demonstrated 95% concordance within ± 1 mm for measurements of PPD.

In the case of a peri-implant health situation, plaque control measures were taken, while in cases of peri-implantitis the patient was referred to receive further treatment, in addition to the reinforcement of plaque control measures. In situations of patients with more than one implant affected by peri-implantitis, the one with the greatest bone loss was chosen for the study.

### Genetic analysis

The following genetic polymorphisms were analyzed: IL-1A-889 and IL-1B+3954 from patients with peri-implantitis and peri-implant health. At position +3954 of exon 5 of the IL-1B gene as well as at position −889 of promoter region of the IL-1A gene there were described single nucleotide polymorphisms (SNPs), which are characterized by replacing a cytosine base (C) with a thymine (T), resulting in two possible alleles: C allele and T allele [25]. For the investigation of genetic polymorphisms, a sample of cells from the jugal mucosa was collected with the aid of a sterile swab (Omniswab Whatman^®^ FTA^®^). Samples were placed in a 1.5-ml test tube (Eppendorf tubes^®^) and kept at −20 °C until DNA extraction. The genetic analysis was done at Egas Moniz Molecular Biology Laboratory with the DNA extraction technique followed by polymerase chain reaction (PCR) and restriction fragment length polymorphism (RFLP). The DNA extraction process was performed based on the “Protocol: Isolation of Total DNA from Surface and Buccal Swabs” taken from the QIAamp^®^ DNA Investigator Handbook.

The DNA obtained was amplified using the PCR technique, and specific primers were used for each of the genes intended to be studied. Table 1 exhibits the sequences of the sense and antisense primers, that is, of the forward and reverse primers, which were described by Kornman et al. (1997).

PCR techniques were performed as described by Kornman et al. (1997). The PCR amplification was performed according to the protocol of the manufacturer of the commercial kit NZYTaq II 2x Green Master Mix^®^. This commercial kit relates to a premixed solution, containing the Taq DNA polymerase enzyme NZYTaq II (0.2 U/ µL), 5 pmol of each primer, 10× PCR reaction buffer, contains 0,5 mM deoxyribonucleotides (dNTPs) and 2.5 mM of MgCl2. PCR reaction occurred with a final volume of 25 µL, containing approximately 50 ng of genomic DNA.

The amplification reactions were carried out in a thermocycler (MJ MINITM Personal Thermal Cycler). For the IL-1A gene polymorphism at position −889, a cycle of 96 °C for 2 min was performed, followed by 45 cycles of 94 °C for 1 min, 50 °C for 1 min and 72 °C for 1 min. Finally, a cycle of 72 °C was carried out for 5 min. For the IL-1B gene polymorphism at position +3954, a cycle of 94 °C for 3 min was performed in a first step, followed by 35 cycles of 94 °C 30 s, 54 °C 35 s and 72 °C 30 s. Afterwards, a cycle of 72 °C was carried out for 5 min.

After PCR amplification, PCR products were restricted with endonuclease NcoI (20,000 U/mL) (BioLabs). The restriction endonuclease was added to PCR products in a 30 µl reaction volume and consisting of 20 µl PCR products, 3 µl 10× NcoI buffer (BioLabs), 1 µl NcoI enzyme, 6 µl of nuclease-free water and were incubated at 37 °C for 16 h. The restriction fragments were visualized with ultraviolet light in the transilluminator (UltraLum). The restriction endonucleases and expected fragments are presented in Table 2.

**Table 1:** Sequence of primers chosen for each of the genetic polymorphisms studied.

Primers	Sequence
IL-1A-889 (Primer Forward)	5′-AAGCTTGTTCTACCACCTGAACTAGGC-3′
IL-1A-889 (Primer Reverse)	5′-TTACATATGAGCCTTCCATG-3′
IL-1B+3954 (Primer Forward)	5′-CTCAGGTGTCCTCGAAGAAATCAAA-3′
IL-1B+3954 (Primer Reverse)	5′-GCTTTTTTGCTGTGAGTCCCG-3′

The resulting fragments were separated by size in 5% MetaPhor agarose gel electrophoresis. After enzymatic digestion, 5 µl of DNA loading dye (Bromophenol blue 0.05%) were added to the samples. 15 µl of marker and DNA were placed in the wells. We used the molecular weight marker NZYDNA ladder VI and pUC19 / MspI (HpaII). The electrical current at 100 mA was established for performing the electrophoresis. For visualization and documentation of the agarose gel, an ultraviolet light transilluminator and a polaroid camera were used.

This procedure was repeated for the IL-1B+3954 polymorphism, with the difference that in this case, the restriction enzyme TaqI and the CutSmart^®^Buffer were used and the samples were stored at 65 °C overnight.

### Statistical analysis

Data were submitted to descriptive and inferential analysis methodologies. A 5% significance level (*p* ≤ 0.05) was established in the later case. The prevalence of IL-1A and IL-1B polymorphisms in patients with peri-implantitis and healthy ones was compared by using appropriate inferential statistics methodologies (bivariable analysis/association tests, Chi-square (*χ*2) and Fisher’s exact test), taking into account several sociodemographic and clinical variables: sex, smoking status and history of periodontitis. The median age was compared between groups by using Mann-Whitney test. Statistical analyses were performed using the software IBM SPSS Statistics v.27.

**Table 2:** Relation of restriction endonucleases, temperature and expected fragments.

Gene	Restriction endonucleases	Restriction profile	Temperature (°C)	Fragments
IL-1A^−889^	NcoI	5′-C/CATGG-3′ 3′-GGTAC/C-5′	37 °C	83 bp + 16 bp (allele 1) 99 bp (allele 2)
IL-1B^+3954^	Taql	5′-T/CGA-3′ 3′-AGC/T-5′	65 °C	12 bp + 85 bp+ 97 bp (allele 1) 12 bp +182 bp (allele 2)

## Results

### Demographic and clinical characteristics of the study group

The study included 20 healthy subjects (nine men and 11 women) ages 32 to 79 years (median: 57 years) who had at least one dental implant inserted. All participants had the same ethnical origin (Caucasian). Factors examined for prognostic significance included cigarette smoking and history of periodontitis. The characteristics of the sample are presented in Table 3.

The percentage of smokers was 30% in the peri-implantitis group, while none of the patients in the peri-implant healthy group was classified as smoker. In addition, positive history of periodontitis was more frequent in the peri-implantitis group than in patients with peri-implant health, albeit the difference in proportion was not significant (80% *vs* 50%; Fisher’s exact test, *p* = 0.35).

### Allelic frequencies

Regarding the IL-1A gene polymorphism, in the health group the allele frequency for the C allele was 85% and for the T allele it was 15%. In the disease group, the allelic frequencies were 70% and 30%, for the C and T allele, respectively. Considering the total sample of alleles (*n* = 40) present in this polymorphism, for the 20 patients, there were 31 (77.5%) C alleles and nine (22.5%) T alleles. No statistically significant differences in proportions (Fisher’s exact test, *p* = 0.45) were observed between the health and disease groups, when considering the presence of the mutated allele. The allelic frequencies are presented in Table 4.

**Table 3:** Baseline characteristics of patients with dental implants.

	Peri-implant Health (*n* = 10)	Peri-implantitis (*n* = 10)	*P* ^#^
Age^*^	54.0 (22) 32–79	62.0 (14) 36–74	0.47^##^
**Sex, *n* (%)**			
**Male**	6 (60.0)	3 (30.0)	0.37
**Female**	4 (40.0)	7 (70.0)	
**Smoking, *n* (%)**			
**No**	10 (100.0)	7 (70.0)	_____
**Yes**	0 (0)	3 (30.0)	
**History of Periodontitis, *n*(%)**			
**No**	5 (50.0)	2 (20.0)	0.35
**Yes**	5 (50.0)	8 (80.0)	

**Notes.**

* Presented as medium (IQR), min–max.

# Fisher’s exact test.

## Mann–Whitney test (*U* = 40.5, *p* = 0.47).

**Table 4:** Allelic frequencies of polymorphisms of IL-1A-889 and IL-1B+3954 in the peri-implant health group and in the peri-implantitis group.

Alleles, *n* (%)	Peri-implant health (*n* = 20)	Peri-implantitis (*n* = 20)	Total (*n* = 40)	*p*
IL-1A (-889)				
C	17 (85)	14 (70)	31 (77.5)	0.45
T	3 (15)	6 (30)	9 (22.5)	
IL-1B (+3954)			
C	18 (90)	13 (65)	31 (77.5)	0.13
T	2 (10)	7 (35)	9 (22.5)	

Regarding the IL-1B gene polymorphism, in the health group the allele frequency for the C allele was 90% and for the T allele it was 10%. In the disease group, allelic frequencies were 65% and 35% for the C and T allele, respectively. Considering the total number of alleles present in this polymorphism in the 20 patients (*n* = 40), the same number of C and T alleles as those registered for the IL-1A-889 polymorphism were observed. As already seen for the IL-1A gene polymorphism, no statistically significant differences were found in proportions of the IL-1B gene polymorphism, between the health and disease groups, when considering the presence of the mutated allele (Fisher’s exact test, *p* = 0.13).

### Genotypic frequencies

The genotypic frequencies obtained for the genes IL-1A-889 and IL-1B+3954, in the peri-implant health group, in the peri-implantitis group and for the total sample are presented in Table 5.

Regarding the IL-1A-889 polymorphism, of the ten patients in the healthy group, seven (70%) had the CC genotype, while the remaining three (30%) showed the presence of the CT genotype. In the peri-implantitis group, of the ten patients who are part of it, four (40%) had a CC genotype and six (60%) a CT genotype. The TT genotype was not observed in any of the groups. Analyzing the data obtained for the total sample, in the 20 patients involved in the study, approximately 55.0% had a CC genotype and 45.0% had a CT genotype. No statistically significant differences were found between the health and disease groups, when considering the proportion of the altered genotype (Fisher’s exact test, *p* = 0.37).

Regarding the IL-1B+3954 polymorphism, in the healthy group, the genotypic frequencies were 80.0% for the CC genotype, 20.0% for the CT genotype and 0% for the TT genotype. For the group with diagnosed peri-implantitis, a frequency of 40.0% for the CC genotype, 50.0% for the CT genotype and 10% for the TT genotype was obtained. Considering all individuals involved in the study, the observed genotypic frequencies were 60.0% for the CC genotype, 35.0% for the CT genotype and 5.00% for the TT genotype. For this polymorphism, it was not possible to perform the inferential analysis.

### Characterization of the sample according to the presence of the positive genotype

Of the ten patients in the healthy group, only one (10%) was genotype positive, while the remaining nine (90%) didn’t have this type of genotype. In the peri-implantitis group, of the ten patients that constitute it, five (50%) were genotype positive and the remaining five (50%) were genotype negative. Of the six patients who were genotype positive, five belonged to the disease group. Analyzing the data for the total sample (*n* = 20), six (30%) of the patients were genotype positive, while the remaining 14 (70%) were genotype negative. The Table 6 shows the percentage of subjects who were genotype positive (IL-1A-889 allele 2 plus ILB+3954 allele 2).

**Table 5:** Genotypic frequencies in polymorphisms of IL-1A-889 and IL-1B+3954, in the periimplant health group and in the peri-implantitis group.

Genotype, *n* (%)	Peri-implant health (*n* = 10)	Peri-implantitis (*n* = 10)	Total (*n* = 20)	*p*
IL-1A (-889)				
CC	7 (70)	4 (40)	11 (55)	0.37
CT	3 (30)	6 (60)	9 (45)	
IL-1B (+3954)				
CC	8 (80)	4 (40)	12 (60)	
CT	2 (20)	5 (50)	7 (35)	——–
TT	0 (0)	1 (10)	1 (5)	

Albeit the previous highlighted differences, no association (Fisher’s exact test, *p* = 0.14) was found between the presence of the positive genotype and peri-implantitis.

## Discussion

The two most widely studied genetic variations, with respect to a possible relationship with peri-implantitis, are IL-1A-889T and IL-1B +3954T, which both have shown to be associated with changes in gene expression and protein secretion (Liao et al., 2014).

The IL-1A-889 polymorphism is located in the IL-1A promoter region, and the presence of the T allele has been associated with an increase in the transcriptional activity of the gene. The CC genotype has been associated with a decrease in transcriptional activity and, consequently, with lower levels of interleukin-1 *α*, when compared to the TT genotype. IL-1B+3954 is located in exon 5 of the IL-1B gene, and the replacement of the C allele by the T allele will cause an alteration similar to that described above (He et al., 2020).

Montes et al. (2009) in their study, performed a comparison of several studies that analyze the allelic frequencies of IL-1B+3954 polymorphisms in different populations, namely Chinese, Japanese, Italian, Swedish and Brazilian. Analyzing the data obtained for this polymorphism in our study, the frequency of the mutated allele (T allele) in the IL-1B+3954 polymorphism was 22.5% in the total sample, which is close to the frequencies found in these populations. Comparing data from all populations, although with some discrepancies between them, all countries showed a higher prevalence of C allele in the IL-1B+3954 polymorphism, verifying the same trend appears at the global level and not only at the European level (Montes et al., 2009).

According to the study of Kornman et al. (1997), which was the first study to find an association of these polymorphisms with periodontitis, there is a reference that the frequency of the positive genotype is 29.1% in Caucasian individuals from northern Europe. This observed data is in line with what was verified in the present study, in which it was found that 30% of all participants had at least one altered allele in the two genes IL-A and IL-1B, at positions −889 and +3954, respectively. According to Kornman et al. (1997), patients with this genotype were reported to be more likely to present an increase in periodontal tissue destruction.

**Table 6:** Percentage of subjects who were genotype positive (IL-1A-889 allele 2 plus ILB+3954 allele 2).

		Peri-implant health (*n* = 10)		Peri-implantitis (*n* = 10)		Total	
		*n*	%	*n*	%	*n*	%
Genotype positive	yes	1	10	5	50	6	30
	no	9	90	5	50	14	70

In a study conducted by He et al. (2020), with 144 patients with peri-implantitis and 174 controls, aimed to analyze whether there was a relationship between polymorphisms of the TNF-α, IL-1A and IL-1B genes and peri-implantitis in a non-smoking Chinese population. Regarding IL-1A-889 gene polymorphism, in the disease group, the frequency of the C allele was 70.1% and the frequency of the T allele (altered allele) was 29.9%. In the control group, the frequencies were 84.2% and 15.8% for the C and T allele, respectively. Thus, the allelic frequencies observed in that study are in agreement with the allelic frequencies obtained in the present study, in which a frequency of 70% for the C allele and 30% for the T allele in the peri-implantitis group and a frequency of 85% and 15% respectively, in the control group were observed.

With regard to the IL-1B+3954 polymorphism, He et al. (2020) observed that, in the peri-implantitis group, the allelic frequency for the C allele was 68.8% and for the T allele was 31.2%, while in the health group the allelic frequency was 79.9% for the C allele and 20.1% for the T allele. Thus, it appears that these frequencies are similar to those observed in the present study in the disease group, in which a frequency of 65% for the C allele and 35% was observed for the T allele. Comparing both studies, for both IL-1A-889 and IL-1B+3954 polymorphisms, there is a higher percentage of T alleles in the peri-implantitis group and a higher percentage of C alleles in the health group. He et al. concluded that both the IL-1A-889T polymorphism and the IL-1B+3954T polymorphism are associated with an increased risk of developing peri-implantitis (He et al., 2020). In the present study, there was no statistically significant difference between the health group and the disease group, so that there was no association between these polymorphisms with peri-implantitis. However, although there is no association, it appears that there is a possible tendency for individuals with these genetic polymorphisms to be more susceptible to developing peri-implantitis, as patients with this disease have a greater number of mutated alleles (T allele) and of CT and TT genotypes, compared to individuals with peri-implant health.

A more recent study by Saremi et al. (2021), that included 50 patients with peri-implantitis and 89 patients with peri-implant health, in an Iranian population, concluded that there is an association between the IL-1B+3954 polymorphism and peri-implantitis.

In contrast, Melo et al. (2012), in a study carried out with a total of 47 patients, concluded that there was no association of the IL-1B+3954 polymorphism with peri-implantitis.

Liao et al. (2014) carried out a meta-analysis with the aim of studying the relationship between IL-1 polymorphisms (IL-1A and IL-1B) and the risk of failure/complications at the level of dental implants. In a total of 13 studies included, only four of these presented sufficient data regarding the relationship between genotype positive IL-1A-889 and IL-1B+3954 and peri-implantitis. Thus, with the completion of the meta-analysis, it is observed that, in Caucasian European descendants, there is a significant association between the positive genotype IL-1A-889 and IL-1B +3954 with peri-implantitis. A recent meta-analysis found that carriers of positive genotype of IL-1A-889 and IL-1B+3954 had 1.95-fold risk of peri-implant disease (which included implant failure/loss, marginal bone loss and peri-implantitis). The authors concluded that the positive genotype of IL-1 can be used as predictive marker for peri-implant disease (Jin, Teng & Cheng, 2021).

Despite some agreement between the results obtained in the present study and the studies mentioned above, especially with regard to the allelic frequencies verified for the alleles with and without change between the health and disease groups, there were some differences regarding the influence of these polymorphisms in the development of peri-implantitis. These divergences may be related to differences regarding the ethnicity of the groups included. In the present study, Caucasian individuals from southern Europe were included, whereas in the study by He et al. (2020), patients of Asian origin were included and in the study by Saremi et al. (2021), only Iranian individuals participated. The influence of ethnic and racial variations in the frequency of gene polymorphisms in terms of the genetic susceptibility to a specific disease has been reported (Kornman et al., 1997; Armitage et al., 2000). It has been demonstrated that there is low prevalence of the periodontitis-associated IL-1A (+4845) and IL-1B (+3954) gene polymorphisms in Chinese (2.3%) (Armitage et al., 2000) compared with that reported for Caucasians (36%) (Kornman et al., 1997). Thus, it is of great importance to determine the occurrence of the genotypes in specific ethnic groups and not to extrapolate the information derived from one ethnic group to another. To avoid including patients with different ethnic origins in the same sample, in our study, only patients with Caucasian parents and grandparents were included.

There were also differences in terms of smoking habits between the published studies. In the present study, smokers were included, whereas in the study by He et al. smoking patients were not selected (He et al., 2020). According to some studies there is a synergism between smoking (heavy smokers) and IL-1 polymorphisms in the development of peri-implantitis (Feloutzis et al., 2003; Gruica et al., 2004). Furthermore, there are studies that observed an association between smoking and peri-implantitis (Karoussis et al., 2003; Becker et al., 2017; Dreyer et al., 2018). Therefore, this habit must be evaluated, its possible effect on the disease must be taken into account and the groups should be balanced regarding the existence of the smoking habit. However, in some studies there were no data on the smoking habits of the patients (Lachmann et al., 2007; Saremi et al., 2021) and in the studies where this habit was evaluated there are differences in categorization of smokers and non-smokers which can lead to different results. Furthermore, all of the identified studies relied solely on patient-reported information for the assessment of smoking status.

On the other hand, our study is a pilot study with 20 patients, consisting of a convenience sample to test the feasibility of the study, which may not represent the population of Portuguese individuals with peri-implantitis. Based on the results obtained for the alleles (calculation performed for a power of 80%, and an alpha error of 5%) we can do a sample size estimation for IL-1A of 242 patients and for IL-1B of 86 patients, in a Portuguese cohort of Caucasian patients. Analyzing and comparing with the sample size of the remaining studies, in the one by He et al. (2020) a total of 318 patients were included and a total of 139 subjects participated in the study by Saremi et al. (2021). Most studies that assess risk factors for peri-implant diseases are based on convenience samples. When we evaluate the samples of the studies that address the possible relationship between these polymorphisms and peri-implantitis, we observe that there are studies with samples from 29 (Lachmann et al., 2007) to 318 patients (He et al., 2020). Such sampling methods are sensitive to selection bias, particularly in studies of cross-sectional and case-control design. Subjects should ideally be included based on random selection, preferably having being treated in different environments.

Probably with a larger sample, we could observe statistically significant differences, regarding the presence of these polymorphisms, between the health and disease groups. In fact, there was a difference, although not statistically significant, in the frequencies of altered alleles in the IL-1A and IL-1B genes, at positions −889 and +3954 respectively, between the health and disease group.

In our study, positive history of periodontitis was more frequent in the peri-implantitis group than in patients with peri-implant health, albeit the difference in proportion was not significant. In studies where possible risk factors/indicators for peri-implantitis are investigated, it is important to evaluate the existence of a history of periodontitis. Previous studies have demonstrated that peri-implantitis-related biofilm was similar to that of periodontitis, comprising high levels of periodontal pathogens (Shibli et al., 2008). And it has been reported that the microflora present in the oral cavity before implantation determines the composition of the newly establishing microflora on implants (Mombelli et al., 1995), suggesting that patients with a history of periodontitis might be at high risk for peri-implantitis. Although there are several indices for the assessment of periodontal status, some of which particularly suitable for epidemiological studies, in our study the guidelines of the new classification of periodontal and peri-implant diseases (AAP /EFP 2018) were followed. According to the new classification case definitions of periodontitis and periodontal health may be applied in different contexts: patient care, epidemiological surveys and research studies.

The results described in the current scientific evidence are promising regarding the role of genetic factors, such as the studied polymorphisms, in the development of peri-implantitis. However, the number of studies in the literature regarding this association is quite limited, and some of them do not refer to variables that may impact/influence the results obtained (confounding variables), such as the periodontal status of individuals in the sample, their ethnicity and the presence or not of smoking habits. Thus, and due to the inequalities found between studies already published in the literature, in order to have a better understanding of this possible association, it is necessary to create uniform criteria between future studies. Furthermore, studies with larger samples in different ethnic groups are needed to determine the real effect of genetic polymorphisms on this disease. In the future, it will be necessary to carry out more studies in this area, with larger samples, so that it is possible to identify and obtain results with greater accuracy and evidence regarding the relationship of polymorphisms of IL-1A-889 and IL-1B+3954 with the development of peri-implantitis.

## Conclusions

According to the results of the present study, there was no statistically significant difference in proportions of IL-1 gene polymorphisms between the health and disease groups. So within the limitations of the present study we can conclude, there seems to be no association between IL-1A genetic polymorphisms (IL-1A-889) and IL-1B (IL-1B+3954) and the development of peri-implantitis. However, although no (statistically) association was found, it appears that there is a possible tendency for individuals with these genetic polymorphisms to be more susceptible to developing peri-implantitis, as patients with peri-implantitis have a greater frequency of mutated allele (T allele), compared to individuals with peri-implant health. Regarding the allelic and genotypic frequencies found, these are similar to those found in European populations.

Further prospective studies with a larger group of patients will confirm whether the studied polymorphisms can be used for screening patients prior to implant treatment and may possibly help in increasing the implant success rates, through the development of new point of care tests.

##  Supplemental Information

10.7717/peerj.13729/supp-1 Supplemental Information 1 Raw data Distribution of interleukin-1 genotype in the population studied

## Additional Information and Declarations

### Data Availability

The following information was supplied regarding data availability: The raw measurements are available in the Supplementary File.
